# THE DIFFERENCE BETWEEN CYSTATIN C- AND CREATININE-BASED EGFR AND ASSOCIATIONS WITH COGNITION IN THE CHS

**DOI:** 10.1093/geroni/igad104.3769

**Published:** 2023-12-21

**Authors:** Lindsay Miller, O Potok, Dena Rifkin, William Longstreth, Oscar Lopez, Joachim Ix

**Affiliations:** University of California San Diego, San Diego, California, United States; University of California San Diego, San Diego, California, United States; University of California San Diego, San Diego, California, United States; University of Washington, Seattle, Washington, United States; University of Pittsburgh, Pittsburgh, Pennsylvania, United States; University of California San Diego, San Diego, California, United States

## Abstract

The difference (eGFRDiff) between cystatin C-based estimated glomerular filtration rate (eGFRcys) and creatinine based eGFR (eGFRcr) has been shown to be associated with frailty and mortality, but its association with cognition is unclear. Among 4,764 adults aged ≥ 65 from the Cardiovascular Health Study, we analyzed eGFRDiff per 1-SD lower (18.6 mL/min/1.73m2). Cognitive function was evaluated annually using the Modified Mini Mental State Exam (3MS; range 0-100), and the Digit Symbol Substitution Test (DSST; range 0-90); higher scores mean better performance. We used linear regression for cross-sectional analyses, and mixed models for longitudinal analyses adjusted for demographic and clinical variables. The mean age was 72.2 ±, the mean eGFRDiff was 0.64, and mean follow-up was 4.4 ± years. eGFRDiff was not associated with 3MS at baseline. Each SD lower eGFRDiff at baseline was associated with a 0.82 [95% CI: -1.22, -0.42] lower DSST score. In longitudinal analyses each SD lower eGFRDiff was associated with a significant 0.06 [95% CI: -0.11, -0.01] faster decline in 3MS scores and a 0.04 [95% CI: -0.08, -0.01] faster decline in DSST scores over time. In older adults, we found that lower eGFRDiff was associated with lower cognitive scores at baseline, and associated with more rapid decline over time. This suggests that eGFRDiff could be used clinically as a laboratory-based screen to identify older adults in need for cognitive assessment.

